# Distributed Static and Dynamic Strain Measurements in Polymer Optical Fibers by Rayleigh Scattering

**DOI:** 10.3390/s21155049

**Published:** 2021-07-26

**Authors:** Agnese Coscetta, Ester Catalano, Enis Cerri, Ricardo Oliveira, Lucia Bilro, Luigi Zeni, Nunzio Cennamo, Aldo Minardo

**Affiliations:** 1 Department of Engineering, Università della Campania Luigi Vanvitelli, Via Roma 29, 81031 Aversa, Italy; agnese.coscetta@unicampania.it (A.C.); ester.catalano@unicampania.it (E.C.); enis.cerri@unicampania.it (E.C.); luigi.zeni@unicampania.it (L.Z.); nunzio.cennamo@unicampania.it (N.C.); 2 Instituto de Telecomunicações, Campus Universitário de Santiago, Universidade de Aveiro, 3810-193 Aveiro, Portugal; oliveiraricas@av.it.pt (R.O.); lucia.bilro@av.it.pt (L.B.)

**Keywords:** distributed optical fiber sensors, vibration sensors, polymer optical fibers

## Abstract

We demonstrate the use of a graded-index perfluorinated optical fiber (GI-POF) for distributed static and dynamic strain measurements based on Rayleigh scattering. The system is based on an amplitude-based phase-sensitive Optical Time-Domain Reflectometry (ϕ-OTDR) configuration, operated at the unconventional wavelength of 850 nm. Static strain measurements have been carried out at a spatial resolution of 4 m and for a strain up to 3.5% by exploiting the increase of the backscatter Rayleigh coefficient consequent to the application of a tensile strain, while vibration/acoustic measurements have been demonstrated for a sampling frequency up to 833 Hz by exploiting the vibration-induced changes in the backscatter Rayleigh intensity time-domain traces arising from coherent interference within the pulse. The reported tests demonstrate that polymer optical fibers can be used for cost-effective multiparameter sensing.

## 1. Introduction

Distributed optical fiber sensors represent an effective tool to monitor large structures in several fields, such as geotechnical monitoring, civil structures monitoring, aerospace monitoring, etc. In particular, distributed optical fiber sensors based on Rayleigh scattering exploit the refractive index inhomogeneities that naturally occur along the fiber and can operate either in the time domain [1,2,3] or frequency domain [4,5]. In the former case, a probe pulse is injected from one end of the sensing fiber. The changes in the amplitude and phase of the backscattered light induced by external perturbations, such as strain, vibration, or temperature, are detected as a function of time. The time delay from the transmitted pulse to the returned signal reveals the position of the perturbation. On the other hand, the time evolution of the signal received from a fixed location, as excited by consecutive pulses, permits the determination of the variations of the measurand in the case of dynamic sensing. This method, usually referred to as ϕ-OTDR (phase-sensitive Optical Time-Domain Reflectometry), relies on the use of a laser with a coherence length longer than the probe pulse [2]. Vice versa, when the coherence length of the laser is much shorter than the pulse, the method is simply referred as OTDR. The traditional OTDR method only provides information on the continuity of the fiber; i.e., it informs the user of the existence and location of any break or high point-loss. However, when applying the OTDR on a polymer optical fiber, a largely strained section can be identified through the induced reduction of the core diameter and the consequent increase of the backscatter intensity in that section [6]. In particular, Ref. [6] reports the use of two polymer optical fibers: the polymethylmethacrylate (PMMA) step index polymer optical fiber and the graded-index perfluorinated polymer optical fiber (GI-POF). Through a photon counting OTDR device operating at 650 nm, the authors showed that the backscatter power in the PMMA fiber increased by ≈5 dB for a 16% tensile strain, while the backscatter power increased by ≈1.4 dB in the GI-POF for an applied strain of 3%. While PMMA fibers exhibited a more linear response and a higher measurement range than GI-POF, the latter has a lower attenuation, which potentially allows a measurement range up to 500 m. Compared to silica optical fibers, polymer optical fibers tolerate much higher strain levels (more than 40%) [6]. Therefore, they are well-suited in applications such as geotechnical monitoring where large strains are usually involved, especially when the fiber is directly embedded into technical textiles [7,8]. Furthermore, polymer fibers are usually multimode, which implies a higher threshold for non-linear effects, a larger capture fraction of Rayleigh backscattered light, and the potential to avoid signal fading by detecting many spatial modes in parallel [9].

In this work, we use a GI-POF similar to the one used in Ref. [6], with the purpose of localizing and quantifying the tensile strain acting on the fiber. In addition, we use the same fiber in order to detect the vibrations through ϕ-OTDR measurements. In order to perform coherent (phase-sensitive) and incoherent OTDR measurements using the same laser source, a wavelength-scanning scheme was adopted. In brief, the method consists in performing an averaging of the ϕ-OTDR acquisitions over a number of laser wavelengths. This effectively removes the interference-related signal fluctuations which are detrimental in static strain measurements. The proposed method is somewhat similar to the one reported in Ref. [10], in which strain and vibration were simultaneously detected by comparing the patterns of signal for different laser frequencies (for strain measurements) or the signals for a fixed laser frequency (for vibration measurements). The main difference is that the method in Ref. [10] was intended for very small strain measurements (less than 0.0001%) in single-mode silica fibers, while the method proposed here is for large strain measurements (a few %) in polymer fibers. The intended application field of our proposed sensor is mostly the geotechnical engineering field, where large strains are usually involved, and where acoustic signals can be exploited, e.g., as precursors of landslides [11]. To the best of our knowledge, this is the first report of a fully distributed vibration sensor based on a POF, while a quasi-distributed vibration sensor was demonstrated in Ref. [12] based on the inscription of fiber Bragg gratings along a GI-POF. Compared to Ref. [12], our sensor provides sensing all along the GI-POF without requiring the inscription of special structures.

In the following section, we will describe the experimental apparatus used for the tests and discuss the experimental results. Section 3 will follow.

## 2. Experimental Procedure and Results

**Figure 1:**
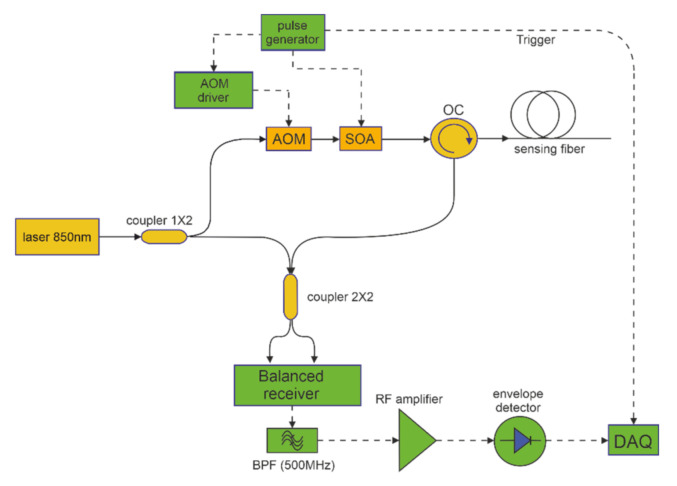
Experimental setup for ϕ-OTDR measurements at 850 nm. AOM: acousto-optic modulator; SOA: semiconductor optical amplifier; OC: optical circulator; BPF: bandpass filter; DAQ: data acquisition system.

The measurements have been carried out using a ϕ-OTDR setup based on heterodyne coherent detection and operating at the wavelength of 850 nm (see Figure 1). The choice of operating at the wavelength of 850 nm mainly derives from the fact that polymer optical fibers suffer from extremely high loss at the conventional wavelength of 1550 nm. Furthermore, the sensitivity of Rayleigh measurements at shorter wavelengths is higher due to the dependance of the Rayleigh scattering coefficient on the inverse of the fourth power of the excitation wavelength [13].

The fiber chosen for the tests is a GI-POF (Fontex-50 from Asahi Glass Company, Tokio, Japan). The optical losses at 850 nm are lower than 70 dB/km, allowing for performing Rayleigh backscattering measurements over a few tens, or even hundreds of meters, depending on the sensitivity of the receiver. The fiber has a core diameter of 55 ± 5 µm, a cladding diameter of 490 ± 5 µm, and a protective jacket diameter of 2 mm. Each end of the GI-POF spool used for the tests was spliced with photopolymerizable resin to a 50-µm step-index silica pigtail fiber, with the splice joint protected using a plastic tube filled with silicone.

The setup is constituted by a distributed feedback (DFB) diode laser emitting at 850 nm and with a linewidth of 3 MHz. The output light is split in two branches by a 50/50 coupler: one of the two branches is used as a local oscillator for coherent detection, while the other one is used to excite the Rayleigh scattering in the GI-POF. The probe light is first pulsed through an acousto-optic modulator (AOM) with a frequency shift of 500 MHz, then amplified through a semiconductor optical amplifier (SOA) up to +13 dBm, and finally injected into the GI-POF through an optical circulator.

The backscatter light is mixed with the local oscillator using a 2 × 2 directional coupler for heterodyne coherent detection. At the receiver end, the 500 MHz intermediate frequency (IF) output from a balanced receiver is filtered, amplified, and down converted to the baseband through an envelope detector. Finally, the baseband signal is digitized and stored for further processing. All the optical components shown in Figure 1 are single-mode at the wavelength of 850 nm. Note that the phase of the backscatter signal could be obtained by digitizing the IF signal at a sampling frequency of (at least) 1 GS/s, as dictated by the Nyquist theorem. Instead, our measurements were carried out by sampling the baseband signal at 250 MS/s, having a sampling point every 40 cm along the GI-POF. Obviously, the envelope detection process removes the phase information, thus only qualitative vibration measurements could be carried out with our setup [1].

**Figure 2:**
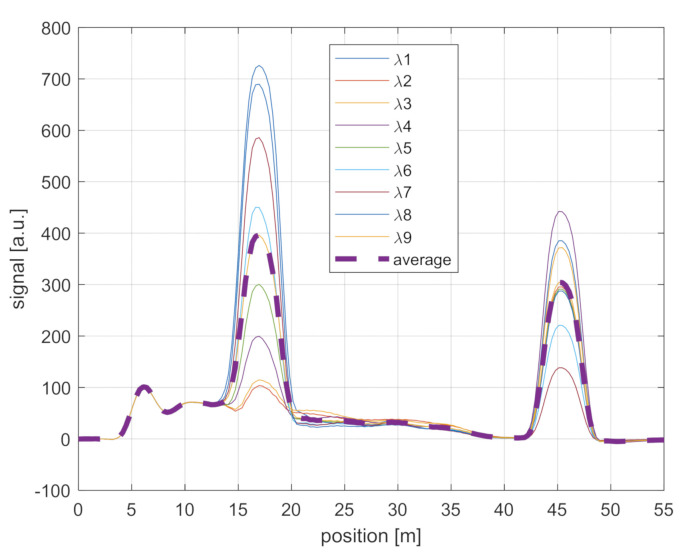
The ϕ-OTDR traces acquired over the GI-POF. The solid lines are the traces acquired for each laser wavelength, while the dashed line corresponds to their average.

As earlier discussed, our ϕ-OTDR scheme has been used to perform both static and dynamic measurements along the same GI-POF. The laser source employed for our tests had a coherence length $L_{c}=c/\pi \Delta \nu \approx 32\;m$, i.e., much longer than the pulse length chosen for our tests (4 m). Thus, the backscatter signal is influenced by the coherent fading noise, which induces spatial fluctuations on the detected signal. These fluctuations are sensitive to any subtle perturbation altering the relative optical phase of the backscatter contributions. While this is required to perform vibration measurements, these changes are detrimental in the case of static measurements since they are based on the increase of the backscatter intensity [6]. In order to get rid of these changes, we have adopted a wavelength-scanning method, in which the ϕ-OTDR measurement is performed over a set of different wavelengths and averaged [14]. As an example, we report in Figure 2 the signals acquired for nine different wavelengths over a GI-POF length of ≈40 m, at a spatial resolution of 4 m. Each trace was the result of $2^{18}$ averages, with the pulse repetition frequency set to 100 kHz. The laser wavelength was tuned by stepping up the DFB temperature by 1 °C (corresponding to a wavelength shift of ≈0.1 nm). We note that the signal acquired at a single wavelength does not show the typical speckle appearance of the ϕ-OTDR signals in a single-mode fiber; i.e., it has a low visibility. This should be attributed to the multimodal propagation in our GI-POF. In fact, multiple modes are excited and detected simultaneously, which averages out the oscillations due to the randomly distributed scatter centers within the injected pulse [8]. We also see that the largest variations in the backward signals occur in correspondence of the Fresnel reflections, one at z ≈ 17 m (probably due to a fiber kink) and another one at the fiber distal end (z ≈ 45 m). The smaller Fresnel reflection at z ≈ 6 m is due to the silica to POF connection.

**Figure 3:**
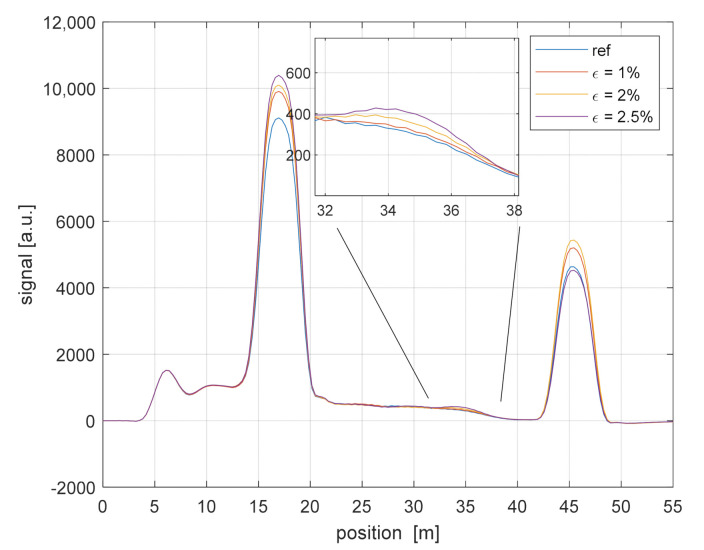
The ϕ-OTDR traces acquired over GI-POF for various strain levels.

The wavelength-scanning method has been applied to detect the local backscatter increase owing to the application of a static strain. We show in Figure 3 the OTDR backscatter signatures recorded while stretching a 2-m GI-POF length up to 2.5% (i.e., extending its length by up to 5 cm). Note that the protective jacket was removed along this GI-POF length before applying tension. The chosen fiber length was stretched by clamping one end to an optical table, while fixing the other end to a linear stage, by which the desired displacement was manually applied. The inset shows the zoom of the output signal in the stretched region, revealing the increase of the backscattered signal with the increase of the applied strain, consistently with the results reported in Ref. [6]. We also see some fluctuations of the signal in correspondence of the Fresnel reflections, which are attributed to the effect of changes in the state-of-polarization of the backward signal over time.

**Figure 4:**
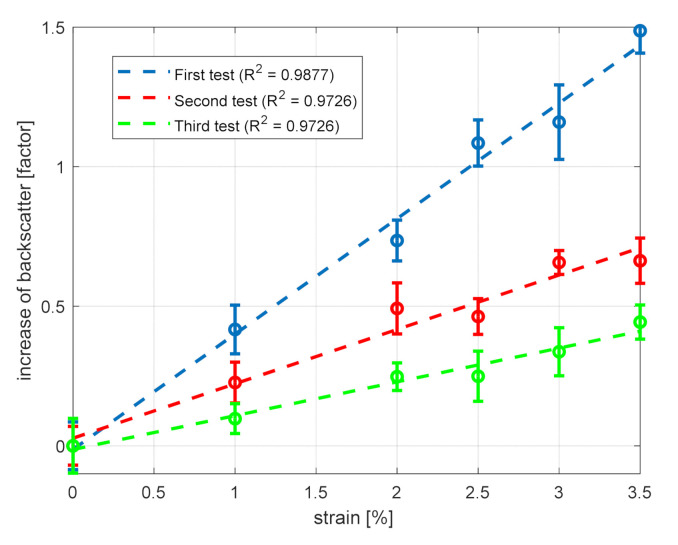
Response of the GI-POF to static strain (circles), and linear fitting curve (lines) over three consecutive tests. The legend also reports the coefficient of determination $R^{2}$ of the linear fits. The error bars represent the standard deviation over 5 consecutive measurements.

Figure 3 also reveals that the optical loss induces a monotonical decay in the OTDR trace, which is superimposed to the changes induced by strain. In order to recover the strain response, while removing the spatial gradients due to the fiber loss, we first calculated the detrended fiber response along the strained region. Then, in analogy with Ref. [6], the backscatter increase was expressed as a factor calculated by integrating the increased backscattering of the strained fiber section relative to the reference measurement and normalizing it to the strained fiber length. This factor of backscatter increase is plotted in Figure 4 (blue circles), together with the result of a linear fit (blue dashed line), for a strain level up to 3.5%. In the same plot, the error bars represent the standard deviation over 5 consecutive measurements. The slope coefficient of the linear fit is 0.41/%, in satisfactory agreement with the results reported in Ref. [6] (in that case, the slope coefficient was ≈0.50/% for strain levels ranging from 2% to 3%).

The retrieved sensitivity can be compared to the theoretical value, as obtained by simple calculations. When a tensile strain is applied to some portion of the GI-POF, the core size (and therefore the mode field diameter, MFD) decreases by a factor related to the applied strain, as well as the Poisson coefficient of the CYTOP material (ν ≈ 0.42 [15]). This leads to a “gain” in the OTDR trace, proportional to the square of the MFD reduction [16]:

(1) $$G={\left(\frac{MFD_{a}}{MFD_{b}}\right)}^{2}$$

where $MFD_{b}\approx MFD_{a}\cdot \left(1-\nu \cdot \varepsilon \right)$ is the MFD of the strained section, $MFD_{a}$ is the MFD of the unstrained fiber, and ε is the longitudinal strain (we are neglecting photoelastic effects). Equation (1) leads to a sensitivity to the strain of the backscatter increase, normalized to the length of the strained section (2 m), equal to 0.42/%, which is in very good agreement with the slope coefficient of the linear fit (0.41/%).

We also estimate the strain resolution by calculating the standard deviation of the acquired traces in the non-strained region (i.e., from z=20.5 m to z=32 m in Figure 4), which is equal to 0.08. From this, the strain resolution is estimated as 0.08/0.41 = 0.2%.

The same experiment was performed two more times over the same fiber section in order to analyze the repeatability of the fiber response to strain, as well as the residual effects after strain removal. In Figure 4, the results of the three characterization tests are compared, together with the corresponding linear fits. Note that the blue squares correspond to the measurements done on the pristine fiber; the red squares represent the results of the test performed immediately after the first one; and the green squares represent the data acquired one day later. We see that the increase of backscatter factor is progressively reduced over the three tests, with the slope of the linear fit being 0.41% in the first test, 0.19% in the second test, and 0.12% in the last test. This can be explained by considering that the backscatter signal in the strained region does not return to the original value after strain removal. In turn, this may be due to the fact that, for strain levels up 3.5%, the fiber enters the plastic regime. Therefore, our tests showed that the percentage increase of the backscatter signal is progressively reduced over successive strain tests. Probably, a thermal recovery cycle could help in restoring the pristine condition as shown in Ref. [17]. However, we did not perform any thermal recovery cycle.

**Figure 5:**
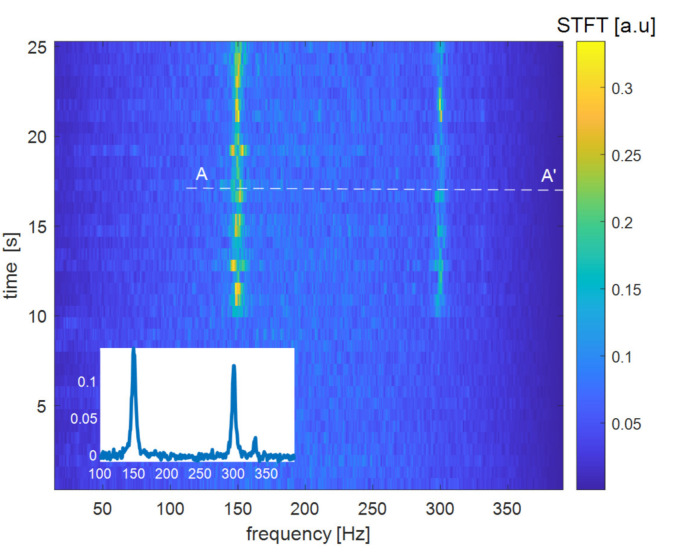
Spectrogram of the ϕ-OTDR signal acquired over the vibrating position. The inset shows the data corresponding to the white dashed line A-A’. STFT stands for short-time Fourier transform.

The next test was aimed at showing the capability to use the same fiber to perform dynamic ϕ-OTDR measurements. To this aim, another 2-m piece along the same GI-POF fiber was wound around a pipe excited by a magnetostrictive actuator and driven at the frequency of 150 Hz. For this test, the ϕ-OTDR trace was acquired at a single, fixed wavelength. The vibrating signal was obtained by taking the difference between the ϕ-OTDR signals over consecutive acquisitions. Figure 5 reports the spectrogram of the signal at the vibrating position obtained by processing a 25-s trace acquired at a sampling frequency of 833 Hz (as resulting from a pulse repetition frequency of 500 kHz and a number of averages of 600). The relatively large number of averages was due to the poor signal-to-noise ratio (SNR) at the detector, which in turn was mostly due to the coupling loss (≈22 dB) between the GI-POF (having a core diameter of 55 µm) and the optical circulator in Figure 1 (having a core diameter of 4.4 µm). In the future, we plan to replace the single-mode circulator with a multi-mode circulator in order to increase the backscatter power collection efficiency and therefore reduce the number of averages. We also note that the vibration was activated 10 s after the beginning of the acquisitions. In Figure 5, the vibration is visible as a peak around 150 Hz. The second harmonic frequency of 300 Hz is visible too, while the third peak at 332 Hz is thought to be due to some external (acoustic) perturbation. We underline that the vibration measurement performed by our amplitude ϕ-OTDR method cannot provide the amount of dynamic strain acting on the fiber. Instead, only the frequency information is retained in the acquired data.

A final test was performed in order to demonstrate the capability of our setup of performing static strain and vibration measurements over the same piece of fiber. To this end, a 2-m long piece of fiber was strained up to 2%. At the same time, the strained fiber was excited acoustically by means of a loudspeaker connected to the audio interface of a PC. The loudspeaker was placed a few centimeters from the middle of the strained fiber and driven with a sine tone at 200 Hz. The measurements were still carried out at a spatial resolution of 4 m.

**Figure 6:**
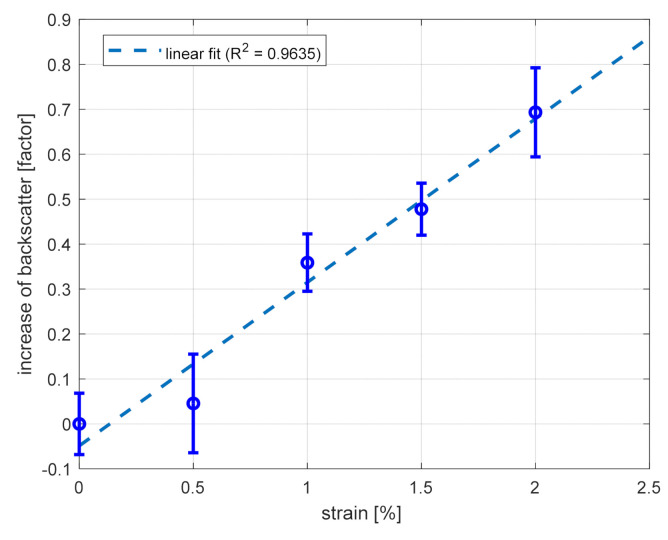
Response of the GI-POF to static strain (squares), and linear fitting curve, as determined during combined strain and vibration measurements. The error bars represent the standard deviation over 5 consecutive measurements.

**Figure 7:**
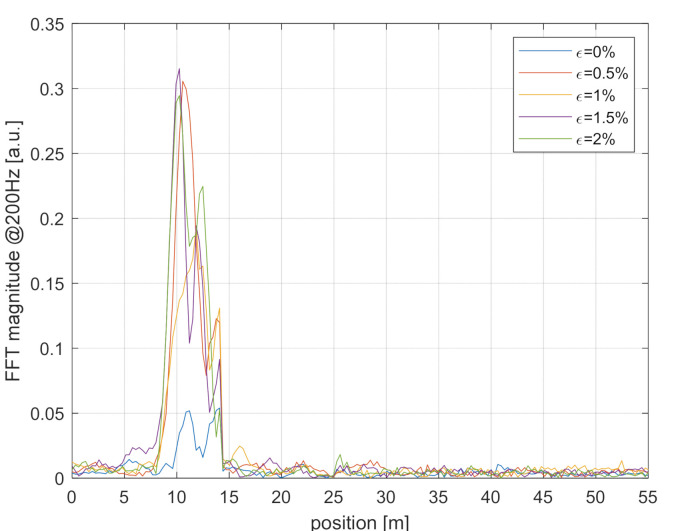
Magnitude of the 200-Hz tone in the acquired ϕ-OTDR signal, as a function of the position and for different static strain levels.

We show in Figure 6 and Figure 7 the results of the static and dynamic measurements, respectively. Regarding the static test, the previously described wavelength-scanning procedure was adopted. The linear fit, shown in Figure 6, gives us an increase of the backscatter factor equal to 0.36/%, in reasonable agreement with the previous test over a pristine fiber region.

The results of the dynamic tests are shown in Figure 7. Each curve represents the magnitude of the 200-Hz component in the backscatter signal, as acquired at the various fiber positions at a sampling frequency of 500 Hz (as resulting from a pulse repetition frequency of 500 kHz and a number of averages of 1000). The acquisition time was 82 s. We observe that the system can localize the acoustic perturbation. Furthermore, we note that the acoustic perturbation is less visible when the fiber is in its loose state (i.e., without any applied tension). This is probably due to a less efficient acoustic coupling with the fiber. Finally, some significant variation of the dynamic signal amplitude is visible within the spatial resolution. This should be attributed to the random changes in sensitivity of the ϕ-OTDR signal to external perturbations, affecting amplitude-based configurations [1].

## 3. Conclusions

We have shown that polymer optical fibers can be used for distributed static and dynamic strain measurements using a ϕ-OTDR setup operating at 850 nm. The static strain was detected by incoherent OTDR measurements at a spatial resolution of 4 m, using a wavelength-scanning method. Vibrations were detected at the same spatial resolution by comparing consecutive ϕ-OTDR traces at a single wavelength. The results demonstrated that polymer optical fibers can be used to detect the vibration and the static strain using a single setup, which can find applications in the geotechnical field. As a future work, the repeatability of the fiber response when subject to large strain cycles must be assessed. Furthermore, we plan to increase the sensitivity of polymer optical fibers to acoustic/vibration perturbations, e.g., by tapering [18] or creating additional scattering centers by damaging the core [19,20], and to enhance the SNR by using multimode components in the setup and adopting a polarization diversity detection scheme.

## Data Availability

Data underlying the results presented in this paper are not publicly available at this time but may be obtained from the authors upon reasonable request.
